# Nanoengineering a metal–organic framework for osteosarcoma chemo-immunotherapy by modulating indoleamine-2,3-dioxygenase and myeloid-derived suppressor cells

**DOI:** 10.1186/s13046-022-02372-8

**Published:** 2022-05-03

**Authors:** Qingxin Fan, Jing Zuo, Hailong Tian, Canhua Huang, Edouard C. Nice, Zheng Shi, Qingquan Kong

**Affiliations:** 1 Hospital of Chengdu Office of People’s Government of Tibetan Autonomous Region (Hospital.C.T.), Sichuan University, Chengdu, 610041 China; 2 State Key Laboratory of Biotherapy and Cancer Center, Collaborative Innovation Center for Biotherapy, Sichuan University, Chengdu, 610041 China; 3 Department of Orthopedics, Hospital of Chengdu Office of People’s Government of TibetanAutonomous Region (Hospital.C.T.), Orthopedic Research Institute, West China Hospital, Sichuan University, Chengdu, 610041 China; 4 Department of Biochemistry and Molecular Biology, Monash University, Clayton, VIC 3800 Australia; 5 Clinical Medical College &, Affiliated Hospital of Chengdu University, Chengdu University, Chengdu, 610106 China

**Keywords:** Osteosarcoma, Nanosystem, Metal organic framework, Chemotherapy, Immunotherapy

## Abstract

**Background:**

The high postoperative recurrence rate and refractoriness of relapsed tumors are still a conundrum for the clinical management of osteosarcoma (OS). New therapeutic options are urgently needed. Depriving the nourishment of myeloid-derived suppressor cells is a novel strategy to improve the immunosuppressive tumor microenvironment for enhanced OS therapy.

**Methods:**

We synthesized a hyaluronic acid (HA)-modified metal–organic framework for combinational chemotherapy and immunotherapy of OS. Zeolitic Imidazolate Framework-8 (ZIF-8) was prepared by a one-pot synthetic method, Gemcitabine (Gem) and D-1-Methyltryptophan (D-1-MT) were loaded into the ZIF-8 during the synthesis process to make ZIF-8@Gem/D-1-MT nanoparticles (NPs). The end product (HA/ZIF-8@Gem/D-1-MT NPs) was obtained by HA modification on the surface of ZIF-8@Gem/D-1-MT NPs. The obtained HA/ZIF-8@Gem/D-1-MT NPs have excellent potential as a drug delivery vector for chemotherapy and immunotherapy in vitro and vivo.

**Results:**

The results indicate that HA/ZIF-8@Gem/D-1-MT NPs were readily taken up by OS cells, and that the Gem and D-1-MT were effectively released into the acidic environment. The HA/ZIF-8@Gem/D-1-MT NPs could efficiently decrease OS cell viability (proliferation, apoptosis, cell cycle, migration and invasion). And HA/ZIF-8@Gem/D-1-MT NPs could reactivate antitumor immunity by inhibiting indoleamine 2,3 dioxygenase and myeloid-derived suppressor cells. Furthermore, animal experiments confirmed that HA/ZIF-8@Gem/D-1-MT NPs could induce intratumoral immune responses and inhibit tumor growth. Additionally, HA/ZIF-8@Gem/D-1-MT NPs have a good safety profile.

**Conclusions:**

Our findings demonstrate that the combination of Gem with D-1-MT brings new hope for the improved treatment of OS, while the generation of the nanosystem has increased the application potential and flexibility of this strategy.

**Supplementary Information:**

The online version contains supplementary material available at 10.1186/s13046-022-02372-8.

## Background

Osteosarcoma (OS) is the most common primary bone tumor in children and adolescents with a global incidence of around 3.4 cases per million people per year [1]. For almost 40 years, in spite of the addition of neoadjuvant chemotherapy combined with surgical resection, the survival rate has not significantly improved and the high recurrence rate is still a conundrum for clinical OS therapy [2]. In particular, due to multiorgan toxicity from chemotherapy, and the refractory nature of tumor recurrence, new therapeutic strategies are urgently needed to improve OS therapy.

Gemcitabine (Gem) is a cytidine nucleoside analog. Its metabolites can both hinder DNA replication and induce cell apoptosis [3]. Currently, Gem plus docetaxel or sirolimus is the second-line treatment in OS, but the therapeutic outcome remains unsatisfactory [1]. Therefore, new therapeutic options are urgently needed.

With a deeper understanding of the tumor ecosystem, immunotherapy is emerging as the fourth pillar of cancer therapy. In the tumor microenvironment (TME), functional immune cells, such as antigen-presenting cells, helper T (Th) cells and cytotoxic T lymphocytes (CTLs), are the workhorses for tumor cell clearance. Tumor cells can cooperate with suppressive immune cells, such as tumor-associated macrophages, myeloid-derived suppressor cells (MDSCs) and regulatory T cells (Treg), to create an immunosuppressive tumor microenvironment to resist functional immune cells [4]. Recently, the immunomodulatory effects of Gem have gradually been evaluated. Gem can promote tumor antigen expression and inhibit the accumulation of MDSCs and Treg cells in TME [5–7]. However, Gem alone is still not sufficient to activate anti-tumor immune response due to a decrease of CD4^+^ and CD8^+^ T cells [5, 8, 9].Thus, we propose combination with an immune checkpoint blockade to boost the immune response in TME, and strengthen the anti-OS effect of Gem.

In the TME, tryptophan (Trp) and kynurenine (Kyn) are important immunomodulatory factors. Trp is a key amino acid for CTLs proliferation and function. Kyn can promote the recruitment of MDSCs and the differentiation of Treg cells to preserve immunosuppressive tumor microenvironment homeostasis [10]. The consumption of Trp and accumulation of Kyn are regulated by indoleamine 2, 3-dioxygenase (IDO), which is a rate-limiting enzyme in Trp/Kyn metabolism [11]. D-1-Methyltryptophan (D-1-MT) is an IDO specific inhibitor, which can independently enhance the function of dendritic cells and promote the proliferation of T cells, despite these cells lacking IDO expression [12]. Based on the encouraging results of preclinical studies, multiple clinical trials have been undertaken [13]. Thus, D-1-MT is an extremely potential immune activator, which may enhance the antitumor effect of Gem in OS. However, the exceedingly short half-life of Gem and poor water solubility of D-1-MT have restricted its clinical use. Therefore, new improved delivery systems are urgently needed to overcome these disadvantages.

**Scheme 1.**
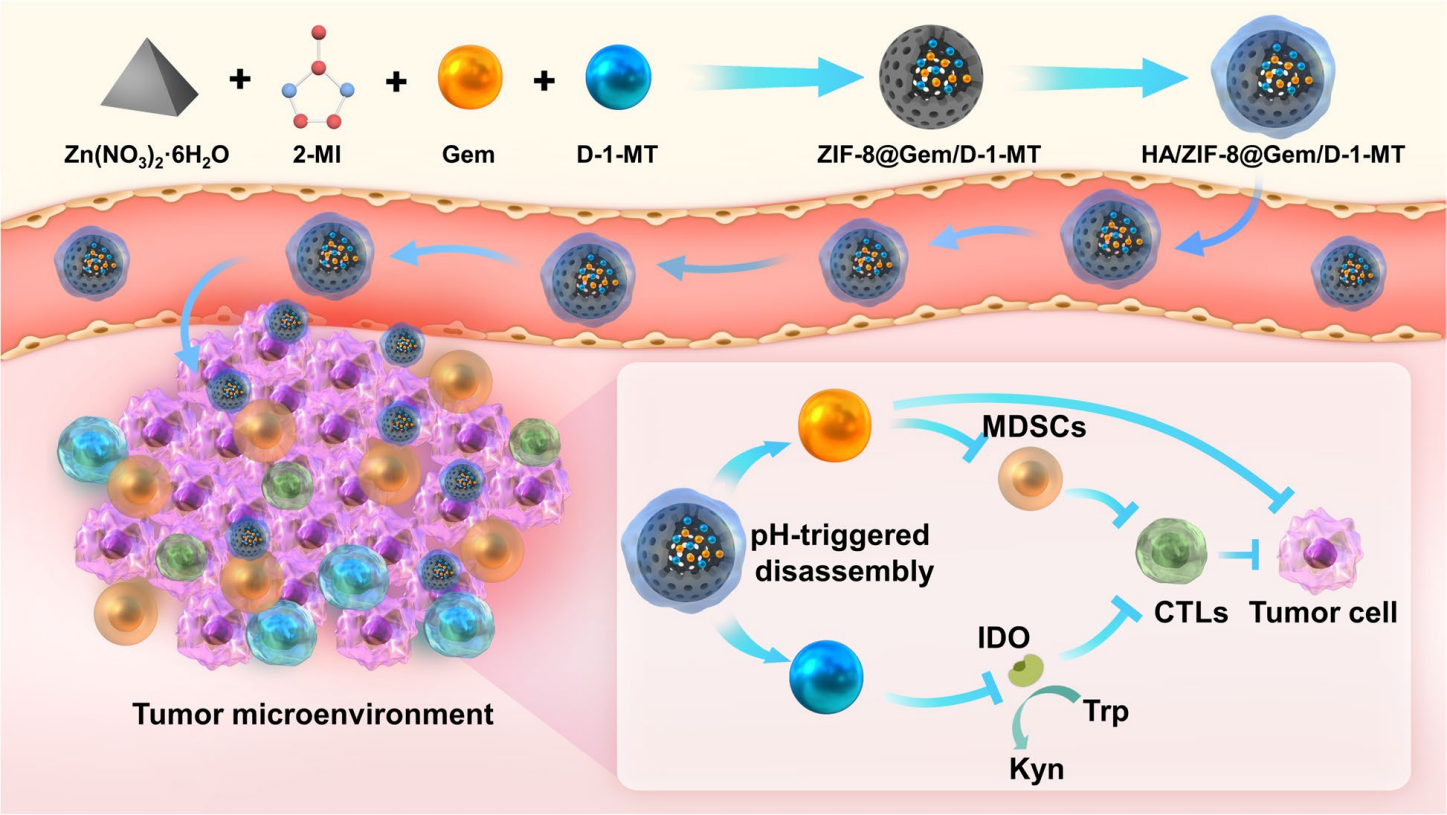
Illustration of the preparation of HA/ZIF-8@Gem/D-1-MT NPs and the mechanisms of the synergistic OS chemo-immunotherapy. 2-MI, 2-Methylimidazole; D-1-MT, D-1-Methyltryptophan; Gem, Gemcitabine; IDO, indoleamine-2,3-dioxygenase; Trp, tryptophan; Kyn, kynurenine; MDSC, myeloid-derived suppressor cell; CTLs, cytotoxic T lymphocyte; DCs, dendritic cells

We have designed a cascaded nanosystem (HA/ZIF-8@Gem/D-1-MT NPs, Scheme 1) to modulate IDO and MDSC for enhanced OS chemo-immunotherapy. Gem and D-1-MT were integrated into the zeolitic imidazolate framework-8 (ZIF-8), resulting in improved solubility of D-1-MT and a longer half-life of Gem. Moreover, hyaluronic acid (HA), a natural polysaccharide, was employed to provide a targeting function [14], while the pH-responsive delivery behavior of ZIF-8 endows the nanoplatform with satisfactory OS accumulation. Mechanistically, Gem can effectively deplete MDSCs while D-1-MT can inhibit IDO in tumor cells and MDSCs to achieve cascaded anti-tumor immunotherapy. Excellent therapeutic effects and immune activation function of the nanosystem were observed in multiple OS cell lines and tumor-xenografted mice. Therefore, the combination of Gem with D-1-MT brings new prospects for the improved treatment of OS, while the generation of the nanosystem has increased the application potential and flexibility of this strategy.

## Materials and methods

### Materials

2-Methylimidazole (2-MI), tetrahydrofuran (THF), trichloroacetic acid, p-dimethylaminobenzaldehyde, L-tryptophan, and acetic acid were obtained from Rhawn Reagent Co., Ltd (Shanghai, China). Zinc nitrate hexahydrate (Zn [NO_3_]_2_·6H_2_O) was obtained from Beijing Bailingwei Technology Co., Ltd (Beijing, China). D-1-MT, Gem and thiazolyl blue tetrazolium bromide (MTT) were obtained from Aladdin Chemistry Co. Ltd (Shanghai, China). Chlorin e6 (Ce6) was obtained from Glpbio Technology Inc. (Montclair, California). HOS and MG63 were obtained from ATCC, K7M2 and MC3T3-E1 were obtained from ProCell (Wuhan, China). DMEM was obtained from Gibco (Gibco, USA). Fetal bovine serum albumin was obtained from Biological Industries (Beit-Haemek, Israel). Crystal violet dye and cell cycle and apoptosis detection kit were obtained from Biyuntian Biotechnology (Shanghai, China). Annexin V-FITC/PI apoptosis detection kit was obtained from Yeasen Biotech (Shanghai, China). IFN-γ was obtained from NovoProtein (Shanghai, China). FITC anti-mouse CD3 Antibody, APC anti-mouse CD4 Antibody, PE anti-mouse CD8a Antibody, FITC anti-mouse CD11c Antibody, APC anti-mouse CD80 Antibody, PE anti-mouse CD86 Antibody, PE/Cy5.5 anti-mouse CD11b Antibody, PE anti-mouse Ly6G Antibody, Alexa Fluor® 488 anti-mouse Ly6C Antibody were obtained from Biolegend (California, USA). Red blood cell lysis solution was obtained from Solarbio (Beijing, China).

### Synthesis of HA/ZIF-8@Gem/D-1-MT NPs

HA/ZIF-8@Gem/D-1-MT NPs were synthesized using the one-pot synthetic method described previously [15] with minor modifications. Briefly, 40 mg of Gem and 40 mg of D-1-MT were dissolved in 4 mL THF (50%) as solution A, 100 mg of Zn (NO_3_)_2_·6H_2_O was dissolved in 0.4 mL double-distilled water (ddH_2_O) as solution B and 1 g of 2-MI was dissolved in 4 mL ddH_2_O as solution C. Solution B was stirred (600 rpm, 5 min) and solution A was added dropwise with stirring for 10 min. Then, the mixture was added dropwise with stirring (800 rpm) to solution C and stirred for a further 15 min. After centrifugation (13,000 rpm, 30 min) and washing with ddH_2_O (3 times), the ZIF-8@Gem/D-1-MT NPs product was obtained. 100 mg of ZIF-8@Gem/D-1-MT NPs was resuspended in 30 mL HA solution (3%, *w/v*), then the suspension was further sonicated using probe sonication for 30 min and magnetically stirred (1000 rpm) for 24 h. After centrifugation and washing with ddH_2_O for 3 times, the HA/ZIF-8@Gem/D-1-MT NPs product was obtained. For bioluminescent imaging, 40 mg of Ce6, 40 mg of Gem and 40 mg of D-1-MT was dissolved in 4 mL THF (50%) as solution D, which was sequentially stirred with solution B and solution C for 15 min. After centrifugation (13,000 rpm, 30 min) and washing with ddH_2_O for 3 times, the HA/ZIF-8@Gem/D-1-MT/Ce6 NPs product was obtained.

### Characterization of HA/ZIF-8@Gem/D-1-MT NPs

The Faraday-Tyndall effect was measured using a 532 nm laser in a dark chamber. The hydrodynamic diameter was measured using a ZetaView PMX110 instrument. The zeta potential of HA/ZIF-8@Gem/D-1-MT NPs were measured with a Malvern Zetasizer Nano-ZS90. The morphology was examined using a Hitachi HT-7800 transmission electron microscope (TEM). The x-ray diffraction (XRD) spectra of HA/ZIF-8@Gem/D-1-MT NPs were obtained with a PANalytical X Pert PRO X-ray diffractometer. NPs were disrupted in HCl solution (pH 1), and the fluorescence intensity measured (Excitation wavelength = 280 nm, Emission wavelength = 344 nm) for D-1-MT and UV absorbance (Wavelength = 271 nm) for Gem to construct appropriate standard curves for detecting drug loading capacity. The release of Gem from HA/ZIF-8@Gem/D-1-MT NPs was carried out using a dialysis method. Briefly, HA/ZIF-8@Gem/D-1-MT NPs were placed into dialysis bags (MWCO = 3500 Da), each dialysis bag containing 1 mL solution. After sealing, all samples were immerged in 49 mL PBS (pH 5.0, 6.8 and 7.4) at 37 °C with rotation at 80 rpm. 1 mL of release medium was then withdrawn and replaced with 1 mL fresh medium at predetermined times. Release of Gem and D-1-MT was calculated from the related standard curves. All assays were conducted in parallel in triplicate.

### Cell culture

The HOS, MG63, K7M2 and MC3T3-E1 cells were incubated in DMEM culture medium with 10% fetal bovine serum albumin and 1% streptomycin-penicillin. The cells were kept at 37 °C and 5% CO_2_ in a moist environment.

### In vitro cell uptake

MG63 and K7M2 were seeded at 2 × 10^5^ cells/well in 6-well plates. At 80% confluence, these cells were incubated with fresh medium containing 10 μM Ce6, ZIF-8@Gem/D-1-MT/Ce6 NPs, or HA/ZIF-8@Gem/D-1-MT/Ce6 NPs at different time points (0 h, 1 h, 2 h, 4 h, 6 h). The same volume of PBS at 0 h was used as a control. After incubation, trypsin was used to digested the adherent cells into single-cell suspensions, and cell pellets were collected by centrifugation (800 rpm, 3 min). Afterwards, cells were washed with pre-cooled PBS to remove medium, and resuspended in PBS (100 μL) for flow cytometric analysis (BD FACSCelesta, USA). 1 × 10^4^ cells were recorded for each sample. Additionally, cells were washed three times for 5 min each after being treated with different formulations, then fixed with 4% paraformaldehyde for 15 min, and the cell images captured using an inverted fluorescence microscope (Nikon, Tokyo, Japan).

### Metabolic activity

A MTT assay was used to evaluate the toxicity and anti-OS effects of various nanosystems. HOS, MG63, and K7M2 cells were chosen as tumor models to evaluate the ability of anti-OS, while MC3T3-E1 cells were selected as normal osteoblasts to study toxicity. Briefly, these cells were plated at 5 × 10^3^ cells per well in 96-well plates. After reaching 80% confluence, cells were treated with D-1-MT or Gem, either freely in solution or in the nanoplatform. After 4 h, the supernatant was discarded, and cells were washed with PBS before the addition of fresh cell medium. After 24 h, a standard MTT assay and data analysis were carried out as reported previously [7].

### Colony formation assay

MG63 and K7M2 were seeded at 2 × 10^5^ cells/well in 6-well plates. At 80% confluence, cells were incubated with fresh medium containing 5 μM D-1-MT, Gem, Gem/D-1-MT, ZIF-8@Gem/D-1-MT NPs, or HA/ZIF-8@Gem/D-1-MT NPs. After 4 h of intervention, the supernatant was discarded, MG63 and K7M2 cells were washed, digested and plated at 600 cells/well in 12-well plates. After 7 days of culture, the adherent cells were fixed with pre-cooled 4% paraformaldehyde for 30 min and then stained with crystal violet dye for 10 min. Cell colonies were counted using ImageJ. Each set of experiments was replicated 3 times.

### Apoptosis

MG63 and K7M2 cells were plated at 2 × 10^5^ cells/well in 6-cm dishes. At 80% confluence, cells were treated with 5 μM D-1-MT, Gem, Gem/D-1-MT, ZIF-8@Gem/D-1-MT NPs, or HA/ZIF-8@Gem/D-1-MT NPs. After 4 h, the medium was renewed, and the cells were cultured for a further 24 h. The suspension and adherent cells were collected and incubated in 100 μL of binding buffer containing 5 μL of Annexin V-FITC and 10 μL PI (Yeasen) for 30 min in the dark. Fluorescence data were acquired on a BD FACSCelesta flow cytometer, and 1 × 10^4^ cells were recorded for each sample.

### Cell cycle assay

MG63 and K7M2 cells were plated at 1 × 10^5^ cells/well in 6-well plates. To synchronize the cell cycle, the medium was replaced with serum-free medium for 24 h when 70%-80% confluent. Following serum starvation, cells were treated with complete medium containing 1 μM D-1-MT, Gem, Gem/D-1-MT, ZIF-8@Gem/D-1-MT NPs, or HA/ZIF-8@Gem/D-1-MT NPs. After 4 h of intervention, the supernatant was replaced with fresh medium after washing three times with PBS. After 24 h, the adherent cells were trypsinized into single-cell suspensions, washed with pre-cooled PBS, fixed with 70% ethanol at 4 ℃ for 2 h, and washed again with pre-cooled PBS. Cells were stained in 500 μL cycle dye solution (*v*: *v*: *v*; dyeing buffer: propidium iodide dye: RNase A; 100: 5: 1) at 37 °C for 30 min, shielded from light. Fluorescence data were acquired on a BD FACSCelesta flow cytometer, and 1 × 10^4^ cells were recorded for each sample. The cell cycle was analyzed using Modfit LT V5.0.

### Migration assay

MG63 and K7M2 cells were seeded at 5 × 10^5^ cells/well in 6-well plates. To reduce cytotoxicity effects in scratch assays, we chose 0.4 μM D-1-MT, Gem, Gem/D-1-MT, ZIF-8@Gem/D-1-MT NPs, or HA/ZIF-8@Gem/D-1-MT NPs as the intervention concentration based on previous MTT results. After the cell confluence reached 90–100%, three parallel linear scratches per well were created using a 200 µL pipette tip, and the nonadherent cells at the scratch edges were washed out with PBS before the intervention started. After 4 h, the medium was replaced with low serum media (1%) and timing commenced. Scratches were photographed at 0, 24, and 48 h on an inverted fluorescence microscope (Nikon, Tokyo, Japan). The cell migration rate was calculated using the gap area method using ImageJ software.

### Invasion assay

25 μL of 10% matrigel (*v*: *v*; Matrigel: DMEM; 1: 9) was evenly spread on the bottom of the upper chamber and left at 37 ℃ for setting. After serum starvation for 24 h, MG63 or K7M2 cells were treated with 0.4 μM D-1-MT, Gem, Gem/D-1-MT, ZIF-8@Gem/D-1-MT NPs, or HA/ZIF-8@Gem/D-1-MT NPs for 4 h, washed by PBS, and seeded at 2 × 10^5^ cells/well into the upper chamber with serum-free media. The upper chamber was then put into a 24-well plate containing 600 μL of complete media. Following incubation for 24 h, the upper chamber was put into 24-well plate containing 600 μL of 4% paraformaldehyde for 30 min. After the non-invading cells were scraped off, the upper chamber was put into a 24-well plate containing 600 μL of crystal violet dye for 10 min. The stained cells were observed and photographed with an Olympus SZX16 Stereomicroscope. Cells were counted using ImageJ software.

### IDO activity assay

IDO enzyme activity in cells was evaluated by measuring the concentration of Kyn [16]. To make the inhibitory effect more visible, 100 ng/mL IFN-γ and 100 μM L-tryptophan were used to enhance IDO enzyme activity [17]. Briefly, K7M2 cells were cultured in transwell chamber with conditioned medium (90% DMEM, 10% fetal bovine serum, 100 ng/mL IFN-γ, 100 μM L-tryptophan). After 80% confluence, cells were treated with D-1-MT (5 μM) or Gem (5 μM), either freely in solution or in NPs form for 72 h. After centrifugation (1000 rpm, 10 min), 1.5 mL supernatant was collected and mixed with 100 μL of 30% trichloroacetic acid at 50℃ for 30 min. Samples were again centrifuged for 1000 min at 2000 × g then 1 mL supernatant was collected and mixed with 1 mL fresh Ehrlich reagent (*v*/*v*; 4-dimethylaminobenzaldehyde: glacial acetic acid; 1:50) at room temperature for 30 min. The absorbance at 492 nm was read on a Synergy H1 plate reader (BioTek).

### Cell sorting

After the mice were killed, the spleens were dissociated to make single-cell suspension. pan T-cells (CD3^+^), Th cells (CD3^+^CD4^+^) and CTLs (CD3^+^CD8^+^) were purified by flow sorting after incubated with CD3-FITC, CD4-APC, and CD8-PE. B cells and T cells in the single-cell suspension were removed by immunomagnetic enrichment with B220 and CD90.2 microbeads for obtaining M-MDSCs and G-MDSCs. Unabsorbed lymphocytes were incubated with CD11b-PE/Cy5.5, Ly6C-Alexa Fluor® 488 and Ly6G-PE. After that, G-MDSCs and M-MDSCs were purified by flow sorting.

### Co-culture experiment

K7M2 cells were treated with 5 μM of D-1-MT, Gem, Gem/D-1-MT, ZIF-8@Gem/D-1-MT NPs, or HA/ZIF-8@Gem/D-1-MT NPs for 4 h or 24 h. After that, the cells were cocultured with purified immune cells in medium (90% RPMI1640, 10% fetal bovine serum, 100 μM L-tryptophan, and 100 ng/mL IFN-γ) at a ratio of 1:10 for 72 h.

### ELISA

The levels of TNF-α and IL-2 in coculture supernatant using the ELISA kits (Shanghai Enzyme-linked, China) following the manufacturer's recommendations.

### Orthotopic OS model

Male BALB/c mice (6 weeks old) were bought from Chengdu YaoKang Biological Technology Co., Ltd. After 1 week acclimatization, mice were anesthetized with 4% chloral hydrate (300 mg/kg). Wells were punched in the marrow cavities of the tibia using a 10 mL syringe needle and filled with 10 µL of K7M2 cell suspension (1 × 10^7^ cells/mL). After the procedure, mice were warmed under a heating lamp until they revived.

### Living imaging

To examine the targeting properties of NPs, 10 mg HA/ZIF-8@Gem/D-1-MT/Ce6 NPs were intravenously injected *in mice* with the equal mass of ZIF-8@Gem/D-1-MT/Ce6 NPs (12 mg) and Ce6 (1.2 mg) used as controls. Mice (*n* = 3 per group) were anaesthetized using 3% isoflurane for induction and 1.5% isoflurane for maintenance and photographed at 1, 2, 4, 8, 24, 72 h post-injection using the IVIS Lumina III system.

### In vivo antitumor efficacy

Mice were randomly divided into six cohorts and treated at 14 d after inoculation (*n* = 4). Briefly, tumor-bearing mice were treated with 200 μL saline solution, D-1-MT, Gem, Gem/D-1-MT, ZIF-8@Gem/D-1-MT NPs or HA/ZIF-8@Gem/D-1-MT NPs at equivalent concentration (20 mg/kg of D-1-MT/Gem) via tail vein injection. Tumor volumes (V) were measured as calculated by the following formula: V (mm^3^) = length × width^2^ × 0.5. The tumor volumes and body weights of mice were recorded every 3 days until day 21 post the start of treatment. Tumors were then removed, weighed and photographed.

### Tumor immune infiltration

The staining procedures of Th cells (CD3^+^CD4^+^), CTLs (CD3^+^CD8^+^), G-MDSCs (CD11b^+^Ly6C^−^Ly6G^+^) and M-MDSCs (CD11b^+^Ly6C^+^Ly6G^−^) were following the manufacturer's instructions. Stained cells were analyzed by flow cytometry using FACScan (BD Biosciences), data were analysed by FlowJo software, 1 × 10^4^ cells were recorded for each sample.

### Safety profile

For histopathological observation, the heart, lungs, liver, spleen, and kidney were removed and repeatedly washed in pre-cooled PBS to clear red blood cells. They were then fixed with 4% paraformaldehyde for 48 h, and embedded in paraffin for H&E staining. For hematological analysis, orbital venous blood was collected and left at room temperature overnight to clot. 150 μL of serum was analyzed for AST, ALT, BUN, and creatinine (Cre) levels.

### Statistical analysis

Statistical analysis was performed using GraphPad prism software. All data were analyzed by one way analysis of variance (ANOVA). Asterisks (*) and/or pound sign (#) denote the statistical significance (*^/#^*P* < 0.05, **^/##^ *P* < 0.01, and ***^/###^ *P* < 0.001).

## Results

### Characterization of HA/ZIF-8@Gem/D-1-MT NPs

**Fig. 1:**
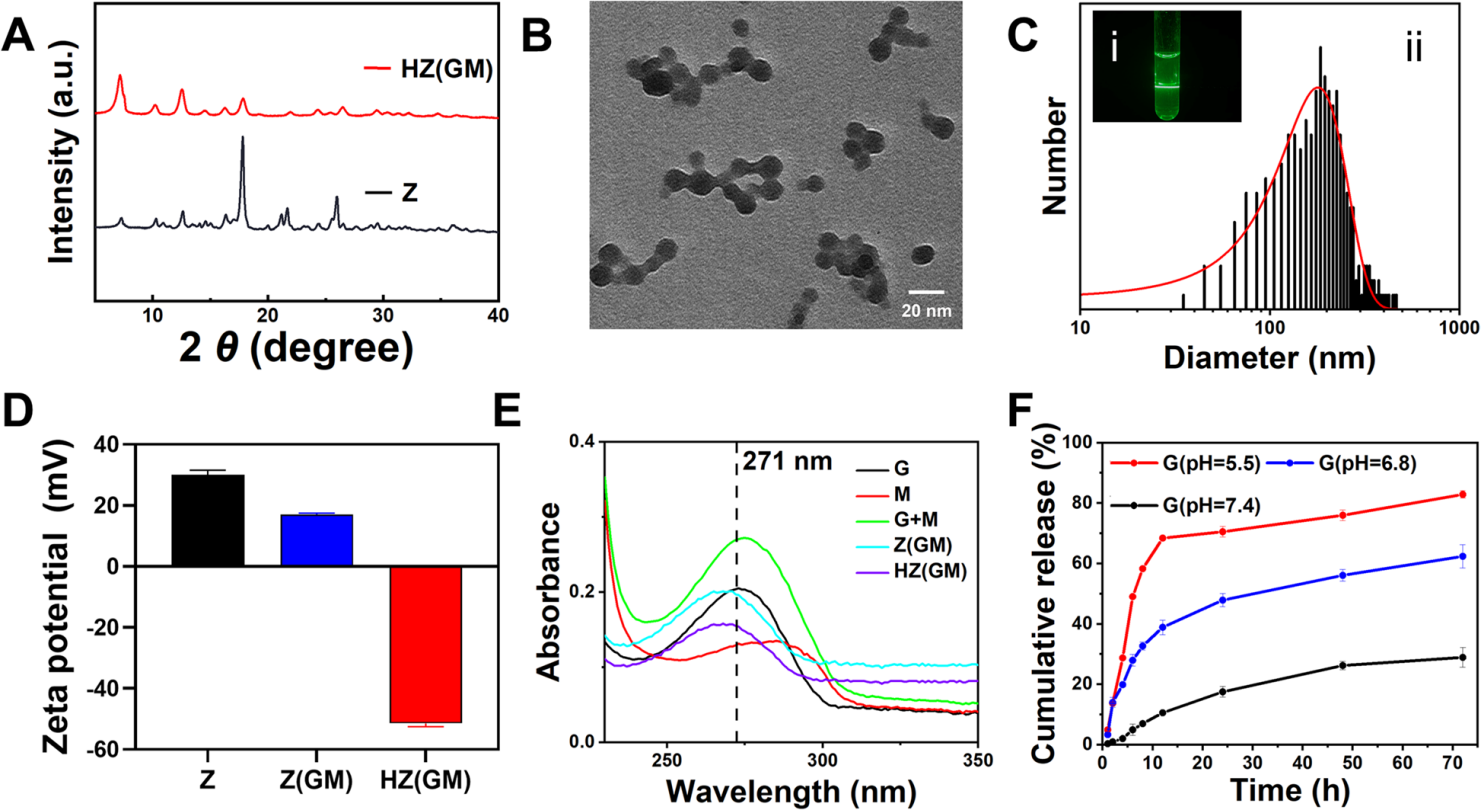
The characteristics of HA/ZIF-8@Gem/D-1-MT NPs. **A** XRD patterns. **B** TEM photograph. **C** Tyndall effect (i) and hydrodynamic diameter distribution (ii). **D** Zeta potential. **E** UV–vis spectra of M, G, M + G, Z(GM), and HA (GM). **F** The release of Gem (*n* = 3). D-1-MT, M; Gem, G; ZIF-8, Z; ZIF-8@Gem/D-1-MT NPs, Z(GM); HA/ZIF-8@Gem/D-1-MT NPs; HZ(GM)

XRD analysis showed the diffraction pattern of ZIF-8 was consistent with previous reports, indicating successful synthesis of ZIF-8 [15]. ZIF-8@Gem/D-1-MT NPs showed similar patterns to ZIF-8 NPs indicating the modification of the preparation method and loading of drugs had not disrupted the crystalline integrity of ZIF-8. However, after HA modification, the HA/ZIF-8@Gem/D-1-MT diffraction peaks displayed a reduced intensity in the low angle range, which might be due to the fact that HA modification on the surface of nanoparticles had a certain masking effect on the crystal structure (Fig. 1A). TEM images HA/ZIF-8@Gem/D-1-MT NPs showed a round homogeneous shape (Fig. 1B). HA/ZIF-8@Gem/D-1-MT NPs exhibited a Faraday-Tyndall effect, suggesting that they could appear as a homogeneous colloid under physiological conditions (Fig. 1C i). Dynamic light scattering analysis showed the hydrodynamic diameter of HA/ZIF-8@Gem/D-1-MT NPs was 195.19 ± 1.84 nm (Fig. 1C ii). The differences between hydrodynamic diameter and electron microscopic diameter might be due to the aggregation between particles, as has been suggested in the TEM photograph (Fig. 1B). However, both the electron microscopic diameter and hydrodynamic diameter data suggested that HA/ZIF-8@Gem/D-1-MT NPs would be suitable for tumor treatment [18]. The zeta potential of HA/ZIF-8@Gem/D-1-MT NPs was -51.47 ± 1.10 mV in PBS which was higher than ZIF-8 NPs (30.07 ± 1.51 mV) and ZIF-8@Gem/D-1-MT NPs (17.03 ± 0.42 mV), indicating the successful HA attachment and HA/ZIF-8@Gem/D-1-MT NPs have excellent stability in physiological environments (Fig. 1D).

To determine the loading capacity, standard curves of Gem and D-1-MT were constructed. The absorbance of HA/ZIF-8@Gem/D-1-MT NPs which had been treated with diluted HCl was recorded (Fig. 1E). The Gem-loading efficiency of ZIF-8@Gem/D-1-MT NPs was calculated as 11.29 ± 0.86% and HA/ZIF-8@Gem/D-1-MT NPs was 8.8% ± 0.57%, while the D-1-MT-loading efficiency of ZIF-8@Gem/D-1-MT NPs was 11.67 ± 0.69% and HA/ZIF-8@Gem/D-1-MT NPs was 9.14% ± 0.84%.

The release of Gem from the NPs was measured at 37 ℃. After 72 h, the release rates of Gem in HA/ZIF-8@Gem/D-1-MT NPs were 28.93% ± 3.21%, 62.41% ± 3.86%, and 82.87% ± 1.22% at pH 7.4, pH 6.8, and pH 5.5, respectively (Fig. 1F). The modification of HA did not affect the release of Gem. For example, under the condition of pH 5.5, Gem could still be released to a 70% level in 24 h. This result indicated that the acidic environment of lysosomes should effectively trigger Gem/D-1-MT release when HA/ZIF-8@Gem/D-1-MT NPs reach the tumor tissue.

### Cellular uptake of HA/ZIF-8@Gem/D-1-MT NPs

**Fig. 2:**
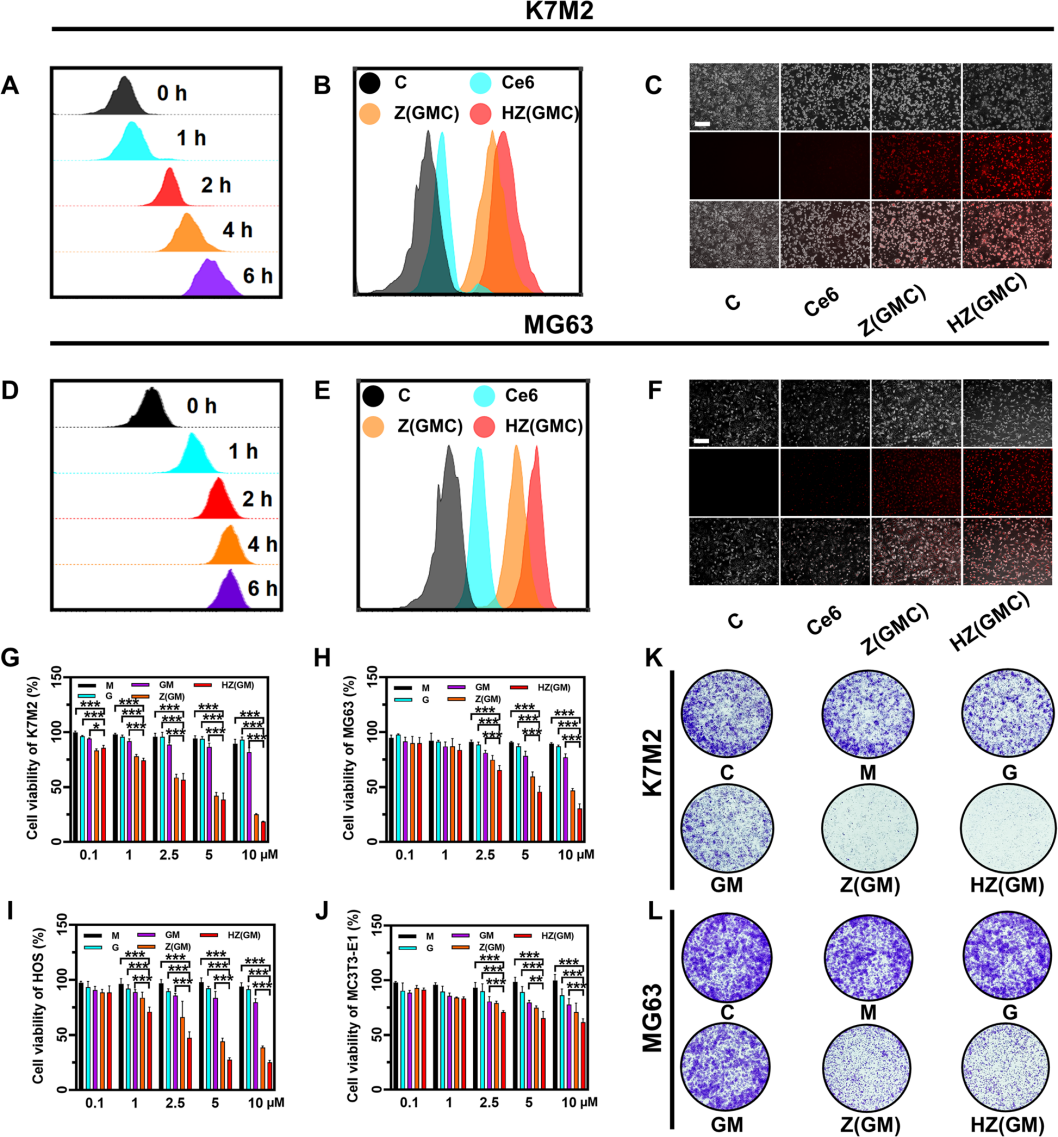
**A** Flow cytometric results of the cellular uptake capacity of HA/ZIF-8@Gem/D-1-MT NPs into K7M2 cells after the incubation at different time periods. Cellular uptake of different formulations into K7M2 cells after incubation for 4 h by flow cytometry (**B**) and inverted fluorescence microscope (**C**) (Scale bar = 200 μm). **D** Flow cytometric results of the cellular uptake capacity of HA/ZIF-8@Gem/D-1-MT NPs into MG63 cells after the incubation at different time periods. Cellular uptake of different formulations into MG63 cells after incubation for 4 h by flow cytometry **E** and inverted fluorescence microscope (**F**) (Scale bar = 200 μm). MTT assay to detect the cytotoxicity of K7M2 (**G**), MG63 (**H**), HOS (**I**), and MC3T3-E1 (**J**) incubated with HA/ZIF-8@Gem/D-1-MT NPs or others. Clone formation assay of K7M2 (**K**) and MG63 (**L**) incubated with HA/ZIF-8@Gem/D-1-MT NPs or others. Control, C; ZIF-8@Gem/D-1-MT/Ce6 NPs, Z(GMC); HA/ZIF-8@Gem/D-1-MT/Ce6 NPs; HZ(GMC). (*n* = 4, * *P* < 0.05, ** *P* < 0.01, and *** *P* < 0.001)

Effective cell internalization is a critical step to effectively treat OS using the designed nanoplatform. Since HA/ZIF-8@Gem/D-1-MT NPs are not fluorescent, we co-loaded Ce6 (a second-generation photosensitiser), Gem, and D-1-MT into HA@ZIF-8 to prepare HA/ZIF-8@Gem/D-1-MT/Ce6 NPs. The cellular uptake of HA/ZIF-8@Gem/D-1-MT NPs was evaluated using flow cytometry and inverted fluorescence microscopy. HA/ZIF-8@Gem/D-1-MT/Ce6 NPs had the ability to enter the cells, reaching a maximum uptake at 4 h in K7M2 (Fig. 2A) and MG63 cells (Fig. 2D). Compared with free Ce6 and ZIF-8@Gem/D-1-MT/Ce6 NPs, the HA/ZIF-8@Gem/D-1-MT/Ce6 NPs treatment group showed stronger fluorescence intensity in both K7M2 (Fig. 2B and C) and MG63 (Fig. 2E and F) cells, indicating that HA/ZIF-8 improve the cellular uptake efficiency.

### In vitro cytotoxicity of HA/ZIF-8@Gem/D-1-MT NPs

The cytotoxicity effects of HA/ZIF-8@Gem/D-1-MT NPs towards OS cells and normal osteoblasts were then evaluated using a MTT assay. HA/ZIF-8@Gem/D-1-MT NPs exhibited significantly higher inhibition effect than the free drug at an equivalent dose in K7M2 cells (Fig. 2G). Similar results were obtained in MG63 (Fig. 2H) and HOS cells (Fig. 2I). Colony formation assays demonstrated that HA/ZIF-8@Gem/D-1-MT NPs decreased K7M2 (Fig. 2K) and MG63 (Fig. 2L) colony formation ability. Since HA and ZIF-8 exhibit only low toxicity [19], this effect was probably due to HA/ZIF-8 increasing the intracellular uptake of the drug. Since osteoblast function is the key to postoperative bone repair in OS patients, we next determined the toxicity of HA/ZIF-8@Gem/D-1-MT NPs to normal osteoblasts in the osteoblastic cell line MC3T3-E1. Compared with OS cells, HA/ZIF-8@Gem/D-1-MT NPs slightly increased the inhibition rate over the free drug at an equivalent dose. MTT assay showed they were able to maintain a low toxicity level over a wide dose range (Fig. 2J).

### Cell apoptosis and cell cycle evaluation

**Fig. 3:**
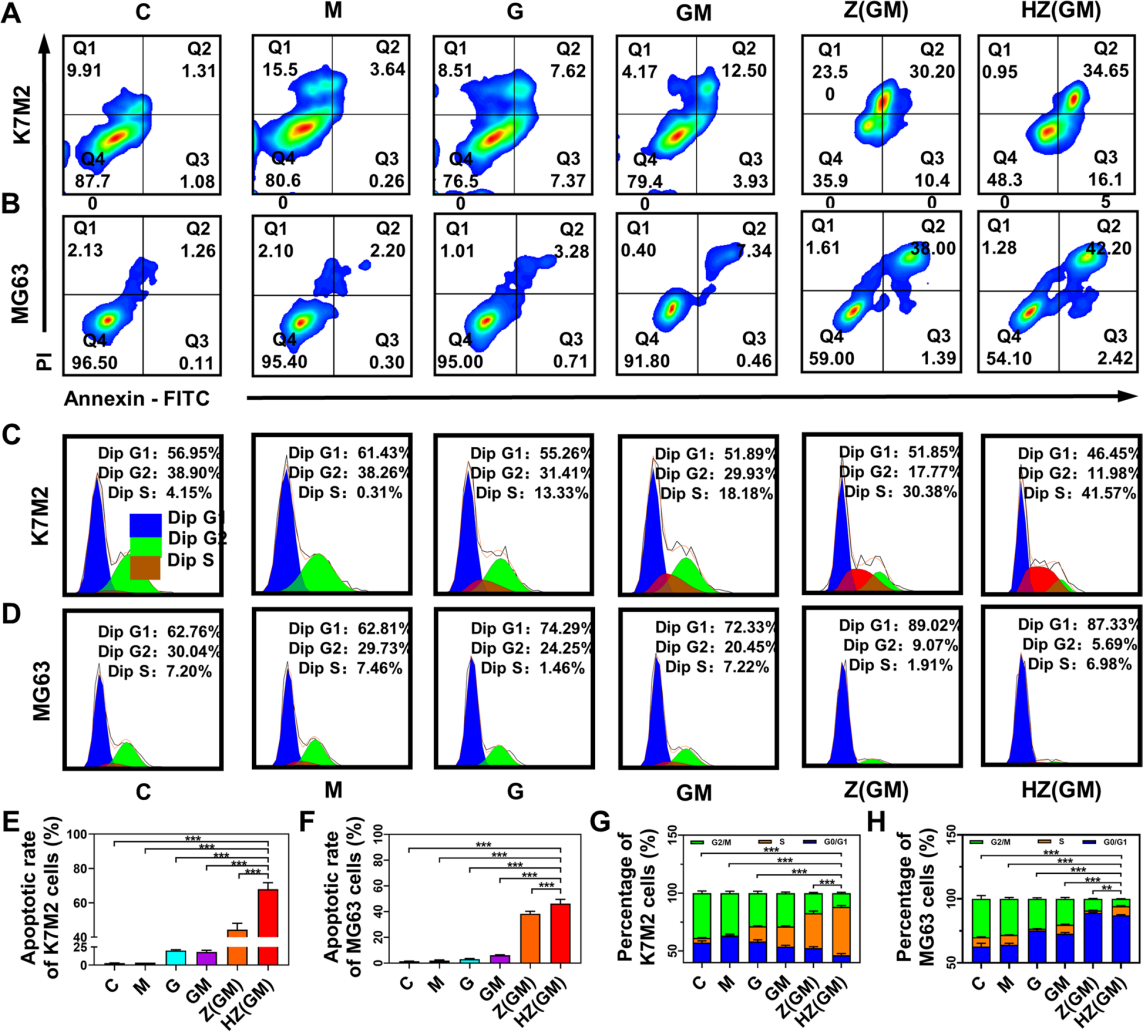
Flow cytometry to detect the apoptosis of K7M2 (**A**) and MG63 (**B**) incubated with HA/ZIF-8@Gem/D-1-MT NPs or others. Flow cytometry to detect the cell cycle of K7M2 (**C**) and MG63 (**D**) incubated with HA/ZIF-8@Gem/D-1-MT NPs or others. Quantification of apoptosis in K7M2 (**C**) and MG63 (D). Quantification of cell cycle in K7M2 (**E**) and MG63 (**F**). Control, C; D-1-MT, M; Gem, G; ZIF-8@Gem/D-1-MT NPs, Z(GM); HA/ZIF-8@Gem/D-1-MT NPs, HZ(GM). (*n* = 3, ** *P* < 0.01, and *** *P* < 0.001)

The induction of apoptosis is an important indicator for evaluating anticancer potential. Annexin-V binding and PI staining were used to verify whether the HA/ZIF-8@Gem/D-1-MT NPs was inducing apoptosis. K7M2 (Fig. 3A and E) and MG63 (Fig. 3B and F) cell apoptosis induced by HA/ZIF-8@Gem/D-1-MT NPs was increased compared with the free drugs and ZIF-8@Gem/D-1-MT NPs. Gem is a cytosine nucleoside derivative, and its metabolites can block cell progression into the G2/M phase by interfering with DNA replication. Propidium staining and flow cytometry were used to measure the DNA content of OS cells at the G1/G0, S, and G2/M phase. The cell-cycle progression of K7M2 (Fig. 3C and G) and MG63 (Fig. 3D and H) were efficiently blocked from G0/G1 to G2/M phase. The above experimental results show that HA/ZIF-8@Gem/D-1-MT NPs can induce OS cell apoptosis and cell cycle arrest, and demonstrate improved anti-OS potential.

### Cell migration and invasion abilities

**Fig. 4:**
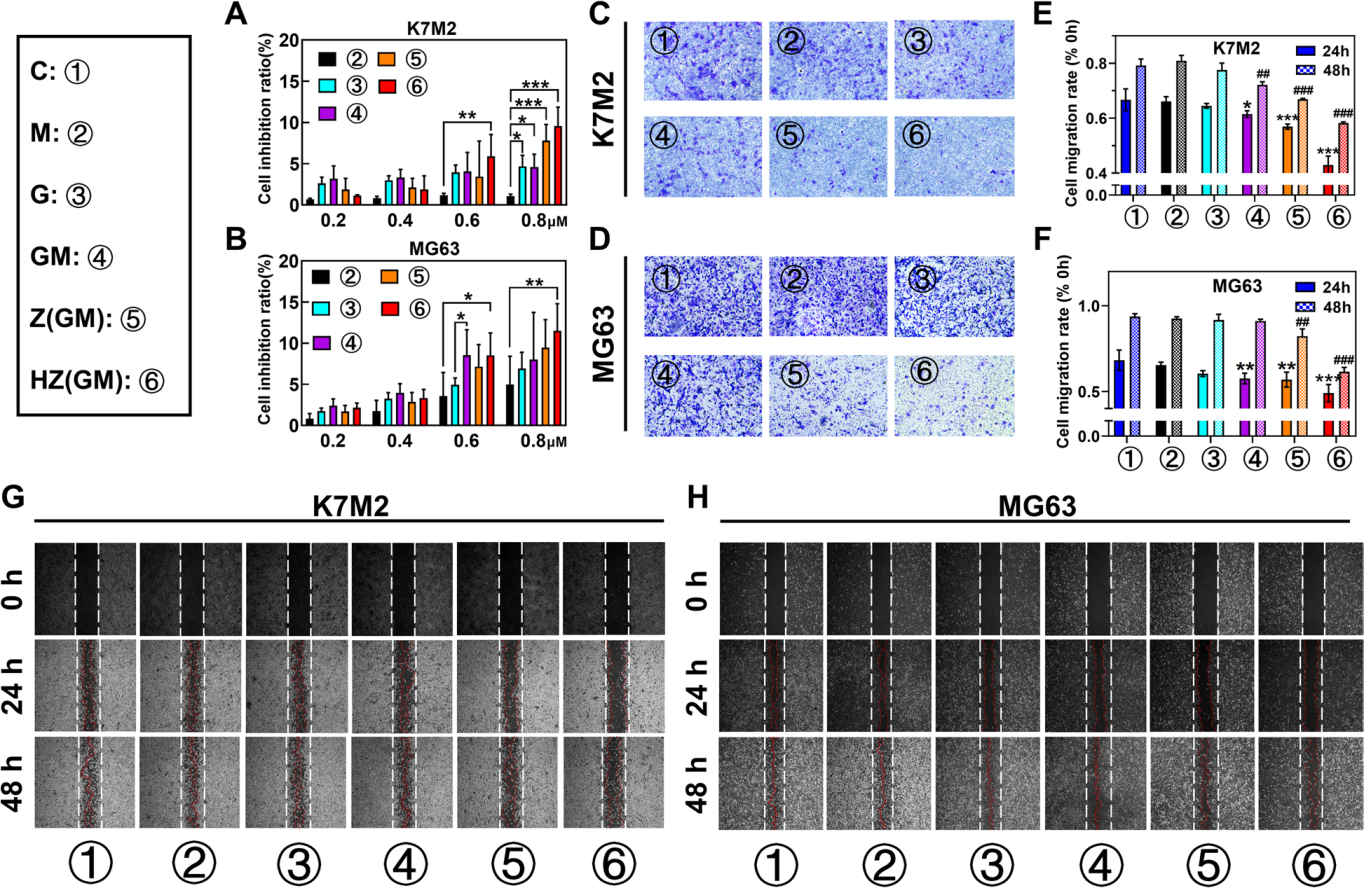
Cytotoxicity of different samples on K7M2 (**A**) and MG63 (**B**) cells. Evaluation of cell invasion of different formulations in K7M2 (**C**) and MG63 (**D**) cells for 24 h. Quantification of migration in K7M2 (**E**) and MG63 (**F**) cells. Evaluation of cell migration ability of different formulations in K7M2 (**G**) and MG63 (**H**) cells for 24 h and 48 h. (*n* = 3, * *P* < 0.05, ** *P* < 0.01, and *** *P* < 0.001 vs. control 24 h; ^**#**^ *P* < 0.05, ^**##**^ *P* < 0.01, and ^**###**^ *P* < 0.001 vs. control 48 h). Control, C; D-1-MT, M; Gem, G; ZIF-8, Z; ZIF-8@Gem/D-1-MT NPs, Z(GM); HA/ZIF, HZ; HA/ZIF-8@Gem/D-1-MT NPs, HZ(GM)

OS is a highly aggressive malignant tumor due to the migration and invasion potential of OS cells. GEM could inhibit migration and invasion by regulating the JAK/STAT [20], HGF/cMET [21], and Akt signaling pathways [22], while D-1-MT mainly inhibited the mTOR signaling pathway [23]. Wound scratch and transwell assays were used to examine the effects of HA/ZIF-8@Gem/D-1-MT NPs on OS cell function. First, to avoid cytotoxicity effects in the wound and transwell assays, MTT was used to screen for low cytotoxicity. Based on MTT data (Fig. 4A and B) to exclude the effect of cytotoxicity, 0.4 μM HA/ZIF-8@Gem/D-1-MT NPs was chosen for subsequent experiments. HA/ZIF-8@Gem/D-1-MT NPs significantly reduced the number of K7M2 (Fig. 4C) and MG63 (Fig. 4D) cells passing through the matrigel, indicating that the invasive ability of the OS cells was severely impaired. Additionally, K7M2 (Fig. 4E and G) and MG63 (Fig. 4F and H) cells treated with HA/ZIF-8@Gem/D-1-MT NPs resulted in a slower wound healing process than other groups. These results showed that HA/ZIF-8@Gem/D-1-MT NPs could reduce the invasion and migration ability of OS cells.

### The mechanism underlying HA/ZIF-8@Gem/D-1-MT NPs-induced immune activation

**Fig. 5:**
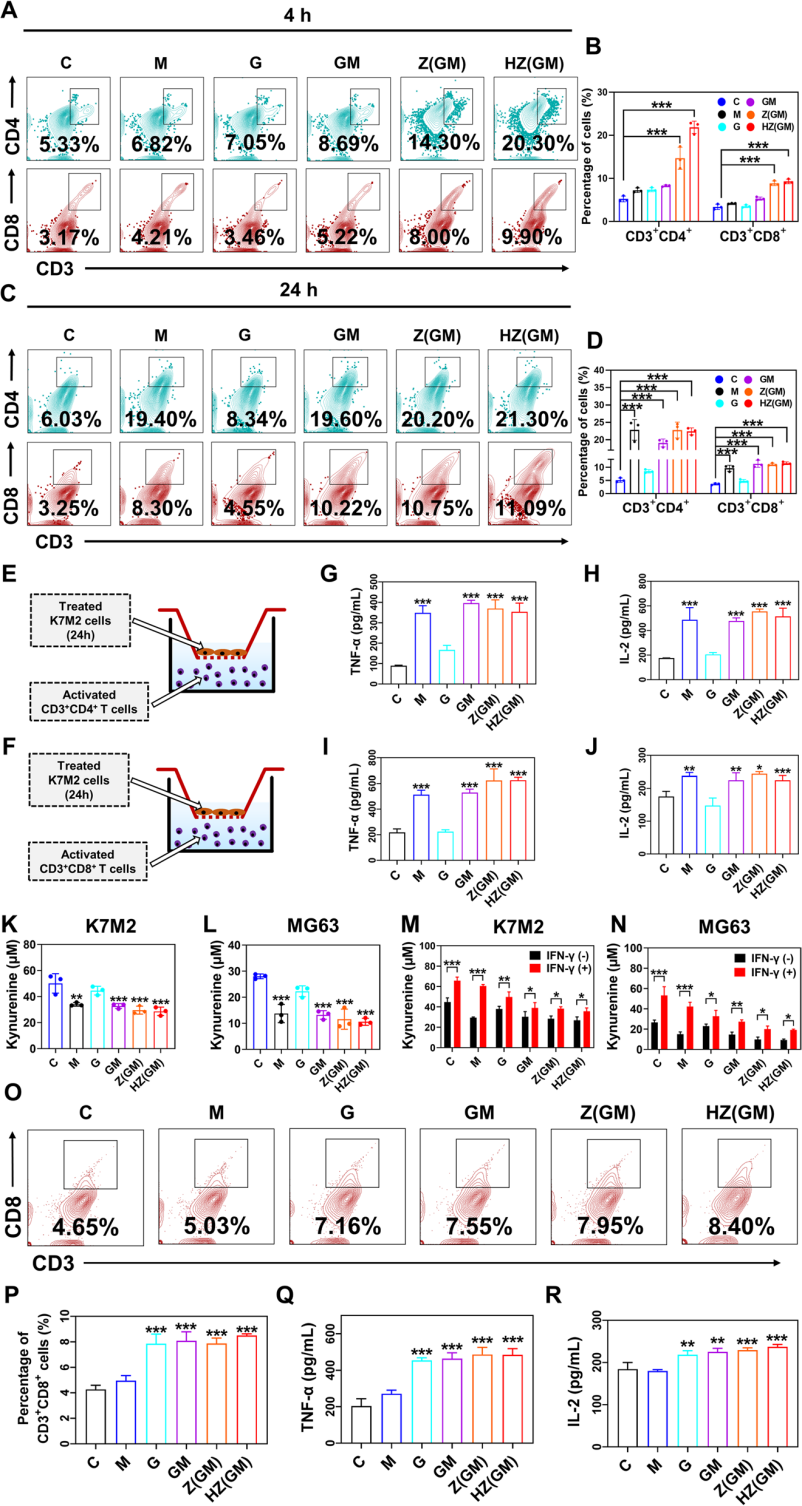
The isolated CD3^+^ T cells were co-cultured with K7M2 cells that were pretreated with indicated treatment for 4 h. After 72 h, the Th cells (CD3^+^CD4^+^) and CTLs (CD3^+^CD8^+^) proportions in the co-culture system were determined by flow cytometry (A-B). The isolated CD3^+^ T cells were co-cultured with K7M2 cells that were pretreated with different formulations for 24 h. After 72 h, the Th cells (CD3^+^CD4^+^) and CTLs (CD3^+^CD8^+^) proportions in the co-culture system were determined by flow cytometry (C-D). Graphic scheme of treated K7M2 co-cultured with Th cells (E) and CTLs (F). (G-H) Measurement of TNF-α and IL-2 levels in the medium of each group. The concentrations of Kyn in the medium were measured following treating K7M2 (K) and MG63 (L) cells with indicated formulation. Change in Kyn concentrations of K7M2 (M) and MG63 (N) cells when treated with or without IFN-γ. The isolated CD3^+^ T cells were co-cultured with MDSCs cells (M-MDSCs: G-MDSCs, 1:2) that were pretreated for 24 h with different formulations. After 72 h, the CTLs proportions in the co-culture system were determined by flow cytometry (O-P). Measurement of TNF-α (Q) and IL-2 (R) levels in the medium of each group. (*n* = 3, *P < 0.01 ** *P* < 0.01, *** *P* < 0.001)

Given the well-established role of IDO in fostering immune tolerance [24, 25], we speculated that pharmacological inhibition of this enzyme might enhance the therapeutic efficacy of Gem-based chemotherapy against OS cells. Therefore, we established appropriate co-culture systems to explore the anti-tumor mechanism of HA/ZIF-8@Gem/D-1-MT NPs. First of all, fluorescent cell sorting analysis was carried out on spleen cells from adult male BALB/C mice through which CD3^+^ T cells were effectively isolated with a purity of > 99% (Fig. S2A-F). Furthermore, the isolated CD3^+^ T cells were then co-cultured with K7M2 cells that were pretreated for 4 h with different formulations (Fig. S1G), after which alterations in the proportion of Th cells (CD3^+^CD4^+^) and CTLs (CD3^+^CD8^+^) subsets were determined and quantified via flow cytometric analysis. Interestingly, incubating K7M2 cells with HA/ZIF-8@Gem/D-1-MT NPs could significantly increase the proportion of Th cells and CTLs from 5.33% to 20.3% and 3.17% to 9.9%, respectively, while no obvious change could be detected in D-1-MT-treated group (Fig. 5A-B). Such a seemingly contradictory result could be simply explained by the observation that agents in nanoparticles were more readily uptaken by OS cells when compared to those in free form (Fig. 2A-F). To address this gap, we cultured K7M2 cells with different formulations for an extended period of time (24 h) to permit enough influx of free compounds into OS cells. As expected, D-1-MT-treated K7M2 cells produced marked increases both in the proportion of Th cells and CTLs, the magnitudes of which were close to those of the HA/ZIF-8@Gem/D-1-MT NPs-treated group (Fig. 5C-D), suggesting a dominant role played by D-1-MT in the enhancement of CTLs infiltration, at least in our co-cultured system.

To further investigate the alterations in T-cell activity, Th cells and CTLs were successfully isolated with purities of 99.4% and 97.9%, respectively (Fig. S3A-I). Using the same co-cultured system (Fig. 5E-F), the sorted Th cells or CTLs were incubated with K7M2 cells following 24-h drug exposure, and then collected supernatant for TNF-α and IL-2 assay. Similarly, treating K7M2 cells with D-1-MT or HA/ZIF-8@Gem/D-1-MT NPs could cause a significant rise of TNF-α and IL-2 production (Fig. 5G-J), indicating that in addition to enhanced infiltration of T cells, the nanoplatform-induced activation of T cells also depended largely on its incorporation of the IDO inhibitor, D-1-MT. Indeed, IDO has been proposed to be the central enzyme that catalyzes the conversion of Trp to Kyn, thus giving rise to an immunosuppressive milieu in tumors via inducing T-cell anergy and apoptosis [24, 26]. In this sense, we were curious whether such a dependence on D-1-MT could also be mechanically linked to the change in the level of this immunosuppressive tryptophan catabolite, and therefore examined the Kyn metabolites following different treatments in K7M2 and MG63 cells. As expected, the levels of Kyn were obviously decreased in groups which contain D-1-MT, especially in HA/ZIF-8@Gem/D-1-MT NPs groups (Fig. 5K-L), and the above phenomenon could be largely restored in OS cells by adding IFN-γ, an IDO agonist (Fig. 5M-N). Together, these observations demonstrated a stimulatory role of HA/ZIF-8@Gem/D-1-MT NPs in T-cell response via D-1-MT-driven inhibition of IDO.

Furthermore, to investigate the possible ability of our nanoplatform in controlling MDSCs-mediated T-cell suppression within the microenvironment of OS, M-MDSCs (CD11b^+^Ly6C^+^Ly6G^−^) and G-MDSCs (CD11b^+^Ly6C-Ly6G^+^) were therefore isolated from spleen cells of adult male BALB/C mice by employing fluorescent cell sorting analysis, and the purities of these two populations were about 99.1% and 99.2%, respectively (Fig. S4A-J). Using the established co-culture system, the already prepared CD3^+^ T cells were incubated with the mixture of M-MDSCs and G-MDSCs that were pre-treated with different treatments for 24 h in a ratio of 1: 2, which could faithfully reflect the in vivo situation (Fig. S4D, K). Consistent with previous studies, the proportion of CD8^+^ tumor-infiltrating lymphocytes exhibited notable increases in response to exposure of Gem- and HA/ZIF-8@Gem/D-1-MT NPs-treated MDSCs when compared to the ones without Gem (Fig. 5O-R). This result indicated that in addition to the inhibition of IDO, our nanoplatform could also enable CTLs response via Gem-mediated modulation of MDSCs.

To summarize, these data suggest that D-1-MT-induced inactivation of IDO together with Gem-mediated modulation of MDSCs can be portrayed as major mechanisms underlying HA/ZIF-8@Gem/D-1-MT NPs-associated T cell response.

### In vivo biodistribution study

**Fig. 6:**
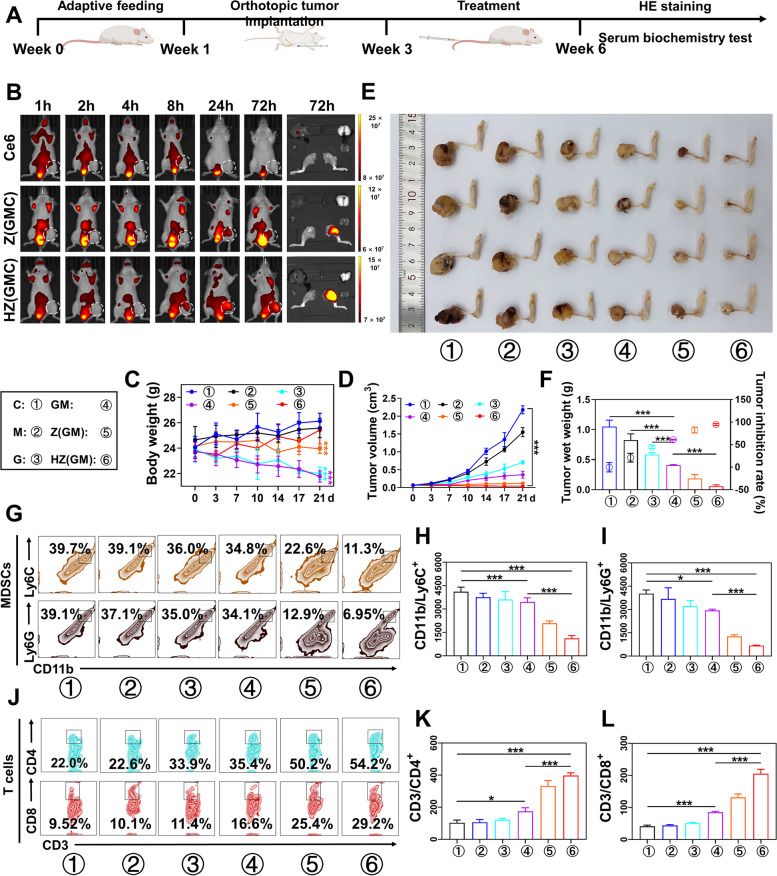
**A** Schematic illustration of the animal experimental model. **B** Bioluminescence images of tumor-bearing BALB/c mice at different time points after intravenous injection with Ce6, ZIF-8@Gem/D-1-MT/Ce6 NPs, and HA/ZIF-8@Gem/D-1-MT/Ce6 NPs. The body weight (**C**) and tumor volumes (**D**) of K7M2 OS-bearing mice with different treatments. (*n* = 4, *** *P* < 0.001). **E** OS tissues were obtained with BALB/c mice on day 21 after receiving different treatments. **F** Weights of isolated tumors and tumor inhibition ratio of K7M2 OS-bearing mice in different groups (*n* = 4, *** *P* < 0.001). **G-I** The percentage of M-MDSCs and G-MDSCs in tumor tissue (*n* = 3, *P < 0.05 and *** *P* < 0.001). **J-L** The percentage of Th cells and CTLs in tumor tissue (*n* = 3, *P < 0.05 and *** *P* < 0.001). Control, C; D-1-MT, M; Gem, G; ZIF-8@Gem/D-1-MT NPs, Z(GM); HA/ZIF-8@Gem/D-1-MT NPs, HZ(GM). ZIF-8@Gem/D-1-MT/Ce6 NPs, Z(GMC); HA/ZIF-8@Gem/D-1-MT/Ce6 NPs: HZ(GMC)

Again, due to a lack of fluorescence characteristics, HA/ZIF-8@Gem/D-1-MT/Ce6 NPs were used to explore the in vivo distribution and metabolism of HA/ZIF-8@Gem/D-1-MT NPs after tail vein injection. Fluorescence imaging showed that HA/ZIF-8@Gem/D-1-MT/Ce6 NPs exhibited excellent tumor accumulation ability, and a stronger fluorescence intensity in the tumor compared with ZIF-8@Gem/D-1-MT/Ce6 NPs (Fig. 6B), suggesting a satisfactory targeting ability of HA. These results indicated HA/ZIF-8@Gem/D-1-MT/Ce6 NPs significant decreased free drug metabolism in the blood circulation, leading to increased drug aggregation in the OS.

### In vivo anti-tumor study

The design of the animal experimental model is shown in Fig. 6A. In situ tumor injection was performed on a BALB/c mice model of orthotopic OS. 14 days after the inoculation of K7M2 cells, the Gem, D-1-MT, Gem/D-1-MT, ZIF-8@Gem/D-1-MT NPs, and HA/ZIF-8@Gem/D-1-MT NPs were injected into the mice via tail vein every two days, using saline as a control. Body weight and tumor size were recorded 14 days after inoculation. Mice were sacrificed 14 days after treatment, and tumors were resected for weighing and photography (Fig. 6A).

Change in body weight is an initial index of safety profile. The body weight of mice in the Gem, Gem/D-1-MT, and ZIF-8@Gem/D-1-MT NPs groups was significantly inhibited. Compared with the controls, the body weight of mice in HA/ZIF-8@Gem/D-1-MT NPs group showed no significant change, maintaining a stable increase (Fig. 6C). This demonstrated the safety of the HA/ZIF-8@Gem/D-1-MT NPs.

All groups exhibited differing degrees of tumor growth inhibition with HA/ZIF-8@Gem/D-1-MT NPs being the most effective (Fig. 6D). Tumor weight and growth rate in the Gem/D-1-MT group was significantly lower than single Gem and single D-1-MT groups, but higher than NPs group (Fig. 6E and F). This result suggested D-1-MT enhanced Gem anti-OS efficacy, but the low bioavailability and non-targeting of free drugs limited antitumor effects. During intervention, the tumor volume of HA/ZIF-8@Gem/D-1-MT NPs group progressively reduced over time until the tumor was virtually effaced. This exciting result indicated the combination of Gem with D-1-MT not only inhibited tumor growth, but also resulted in reversal.

### In vivo immune response

Intratumoral lymphocytes were analyzed to verify the immune status in TME following HA/ZIF-8@Gem/D-1-MT NPs treatment. HA/ZIF-8@Gem/D-1-MT NPs decreased the M-MDSCs and G-MDSCs infiltration in osteosarcoma tissues (Fig. 6G-I). Moreover, Upon HA/ZIF-8@Gem/D-1-MT NPs treatment, intratumoral infiltration of the Th cells and CTLs dramatically raised (Fig. 6J-L). These results indicated HA/ZIF-8@Gem/D-1-MT NPs could effectively turn a poorly infiltrated “cold” tumor into a highly infiltrated “hot” tumor. Additionally, lymphocytes in the spleen were analyzed to verify systemic antitumor immune responses after treatment. It was found that Th cells and CTLs was significantly raised (Fig. S5), which confirming the systemic immune response also was active.

### In vivo safety

**Fig. 7:**
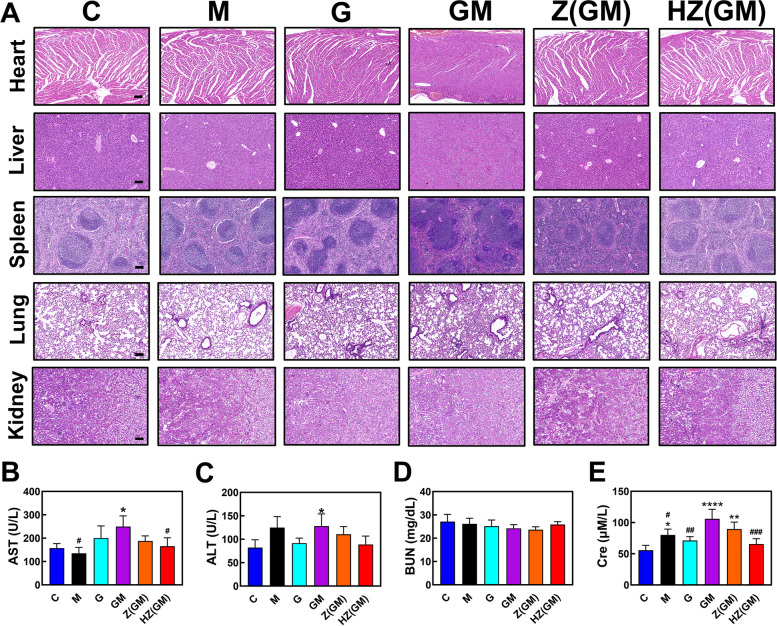
**A** Histological assessments of organs with H&E staining. The scale bar is 100 μm. **B-E** Biochemical analysis of the peripheral blood serum of OS-bearing mice. Control, C; D-1-MT, M; Gem, G; ZIF-8@Gem/D-1-MT NPs, Z(GM); HA/ZIF-8@Gem/D-1-MT NPs, HZ(GM). (*n* = 3, * *P* < 0.05, ** *P* < 0.01, and *** *P* < 0.001 vs. control; # *P* < 0.05, ## *P* < 0.01, and ### *P* < 0.001 vs. Gem/D-1-MT)

To observe potential toxicity of the different treatment strategies, the kidneys, lungs, spleen, liver and heart of tumor-bearing mice were removed and examined using H&E staining (Fig. 7A). In the Gem/D-1-MT group, severe morphological alterations were observed in multiple organs: 1. The cardiomyocytes were significantly enlarged with irregular nucleus and the myofibrils are significantly thicker. 2. The liver showed diffuse degeneration with swollen hepatocytes, unorganized liver cords, and crowded liver sinusoids. 3. The follicle and occurrence center of the splenic lymph nodes were significantly enlarged. 4. The alveolar spaces became narrower with honeycomb-like tracheal epithelium. Compared with the control, there was no obvious damage detected in kidney. Importantly, no obvious changes in any of the organs was detected in the HA/ZIF-8@Gem/D-1-MT NPs group, indicating that intravenous HA/ZIF-8@Gem/D-1-MT NPs could reduce the systemic toxicity of Gem/D-1-MT combination therapy.

Peripheral blood serum was collected and performed for biochemical analysis. Gem/D-1-MT treatment increased AST, ALT, and Cre, indicating that this strategy had slight hepatic and renal toxicity. Compared with the Gem/D-1-MT group, HA/ZIF-8@Gem/D-1-MT NPs treatment significantly reduced the levels of ALT, AST, and Cre. Compared with the control group, there was no significant difference in ALT, AST, BUN, and Cre (Fig. 7B-7E). These results confirm that HA/ZIF-8@Gem/D-1-MT NPs have a good safety profile.

## Discussion

OS is the most common malignant bone tumor, which accounts for 20% of bone tumors, primarily affecting children and adolescents aged 5 to 20 years [27, 28]. Although diverse neoadjuvant chemotherapy has been extensively used in clinical practice to treat OS, the poor prognosis greatly limits their further applications [29], as well as the systemic toxicity and low intratumoral accumulation. Importantly, MDSCs and IDO are the main factors in the OS immune microenvironment that result in immune escape and hinder the treatment of OS [30–32]. MDSCs can produce arginase 1, iNOS, ROS, RNS, TGF-β, IL-10 and induce Tregs for suppressing T cell function [33–36]. IDO can degrade Trp to Kyn in OS cells and MDSCs, which results in the differentiation of the naive CD4^+^ T cells into Tregs and leads to T cell anergy [37–39].

Nanoparticle drug delivery is seeing an increasing uptake in the treatment of cancer and other diseases [40–42]. Nanoparticles have the advantages of stability, high carrier capacity, ability to incorporate both hydrophilic and hydrophobic substances, reduced toxicity, can promote transport across membranes, prolong circulation times, there is the feasibility of variable routes of administration, and they can be designed to allow sustained drug release from the matrix [43, 44]. Thus, integrating nanotechnology with traditional treatments and immunotherapy may largely benefit OS therapy.

In this study, we creatively design a versatile nanoplatform (HA/ZIF-8@Gem/D-1-MT NPs), which can efficiently load chemotherapeutic drugs (Gem) and IDO inhibitor (D-1-MT) for MDSCs depletion and IDO inhibition. The nanoplatform can aggregate in the tumor region through the targeting of HA and EPR effects and subsequently release Gem and D-1-MT in an acidic microenvironment, reducing systemic toxicity. A chemotherapeutic effect on OS cells with consequential MDSCs consumption could be mediated by the released Gem, along with IDO inhibition in OS cells by D-1-MT, resulting in synergistic antitumor immunotherapy. As a result, we observed significant anti-OS effects with the designed nanoplatform in vitro. Moreover, we found that HA/ZIF-8@Gem/D-1-MT NPs can remarkably activate anti-tumor immune responses, the mechanism relates to an inhibition of IDO activity. Consistent with the in vitro results, HA/ZIF-8@Gem/D-1-MT NPs can decreased MDSCs infiltration, increased CD3^+^CD8^+^ CTLs infiltration into the tumor microenvironment, and converted unresponsive “cold tumors” into responsive “hot tumors” for OS treatment. Thus, this provides a safe and effective strategy for enhancing the chemo-immunotherapy of osteosarcoma by inhibiting MDSCs and IDO.

We are currently seeing a paradigm shift in medical treatment as we move from the classical “one size fits all” approach to drug use to one where treatment is optimized based on a comprehensive understanding of personalized systems biology (personalized/precision medicine) [45]. To date, nanomedicine development has focused mainly on optimizing delivery systems with a one-size-fits-all solution [46]. However, continued improvement of this enhanced targeting nanoplatform (HA/ZIF-8@Gem/D-1-MT NPs) in the future can further promote therapeutic outcomes and refine nanoplatforms for the benefit of patients.

## Conclusions

In summary, we have designed a novel chemo-immunotherapy nanoplatform (HA/ZIF-8@Gem/D-1-MT NPs) for the treatment of OS. This nanoplatform improved both Gem half-life and the water solubility of D-1-MT and allows targeted enrichment in the OS microenvironment, indicating the clinical translational potential of this strategy. When HA/ZIF-8@Gem/D-1-MT NPs reach the TME and enter into OS cells, the acidic environment of lysosome will break down the nanoplatform promoting the intracellular release of Gem and D-1-MT. The intracellular metabolites of Gem can block DNA replication, induce OS cell apoptosis and decrease MDSCs. The intracellular D-1-MT can inhibit IDO and block the Trp/Kyn metabolic pathway. Moreover, extracellular Gem can directly inhibit immunosuppressive cells, while D-1-MT can inhibit the IDO activity. This strategy synergizes TME immunity and the direct killing effect to suppress tumor growth. In vitro, OS cells exhibit good uptake and efficient cleavage of HA/ZIF-8@Gem/D-1-MT NPs. HA/ZIF-8@Gem/D-1-MT NPs suppressed OS growth, exerted excellent anti-MDSCs effects and IDO inhibitory effects. In vivo, HA/ZIF-8@Gem/D-1-MT NPs can reactivate anti-tumor immune response and reverse the development of OS. Besides, HA/ZIF-8@Gem/D-1-MT NPs showed good biosafety and significantly decreased the multi-organ toxicity observed with the combination free drug regimen. Therefore, the combination of Gem with D-1-MT brings new hope for the improved treatment of OS, and the generation of the nanosystem has increased the application potential and flexibility of this strategy.

## Supplementary Information

**Additional file 1.**

## Data Availability

All data generated or analyzed during this study are included in the article.

## Abbreviations

2-MI: 2-Methylimidazole
Ce6: Chlorin e6
Cre: Creatinine
CTLs: Cytotoxic T lymphocytes
D-1-MT: D-1-Methyltryptophan
ddH_2_O: Double-distilled water
Gem: Gemcitabine
HA: Hyaluronic acid
HA/ZIF HZ IDO: Indoleamine-2,3-dioxygenase
Kyn: Kynurenine
MDSCs: Myeloid-derived suppressor cells
MTT: Thiazolyl blue tetrazolium bromide
NPS: Nanoparticles; OS: Osteosarcoma
TEM: Transmission electron microscope
Th: Helper T cells
Treg: Regulatory T cells
THF: Tetrahydrofuran
TME: Tumor microenvironment
Trp: Tryptophan
UV-vis: Ultraviolet visible
XRD: X-ray diffraction
ZIF-8: Zeolitic imidazolate framework-8
