# Supplementary figures and images for: Nanoengineering a metal–organic framework for osteosarcoma chemo-immunotherapy by modulating indoleamine-2,3-dioxygenase and myeloid-derived suppressor cells
Source: J Exp Clin Cancer Res. 2022 May 3;41:162. doi: 10.1186/s13046-022-02372-8 (PMC9063269; doi:10.1186/s13046-022-02372-8)

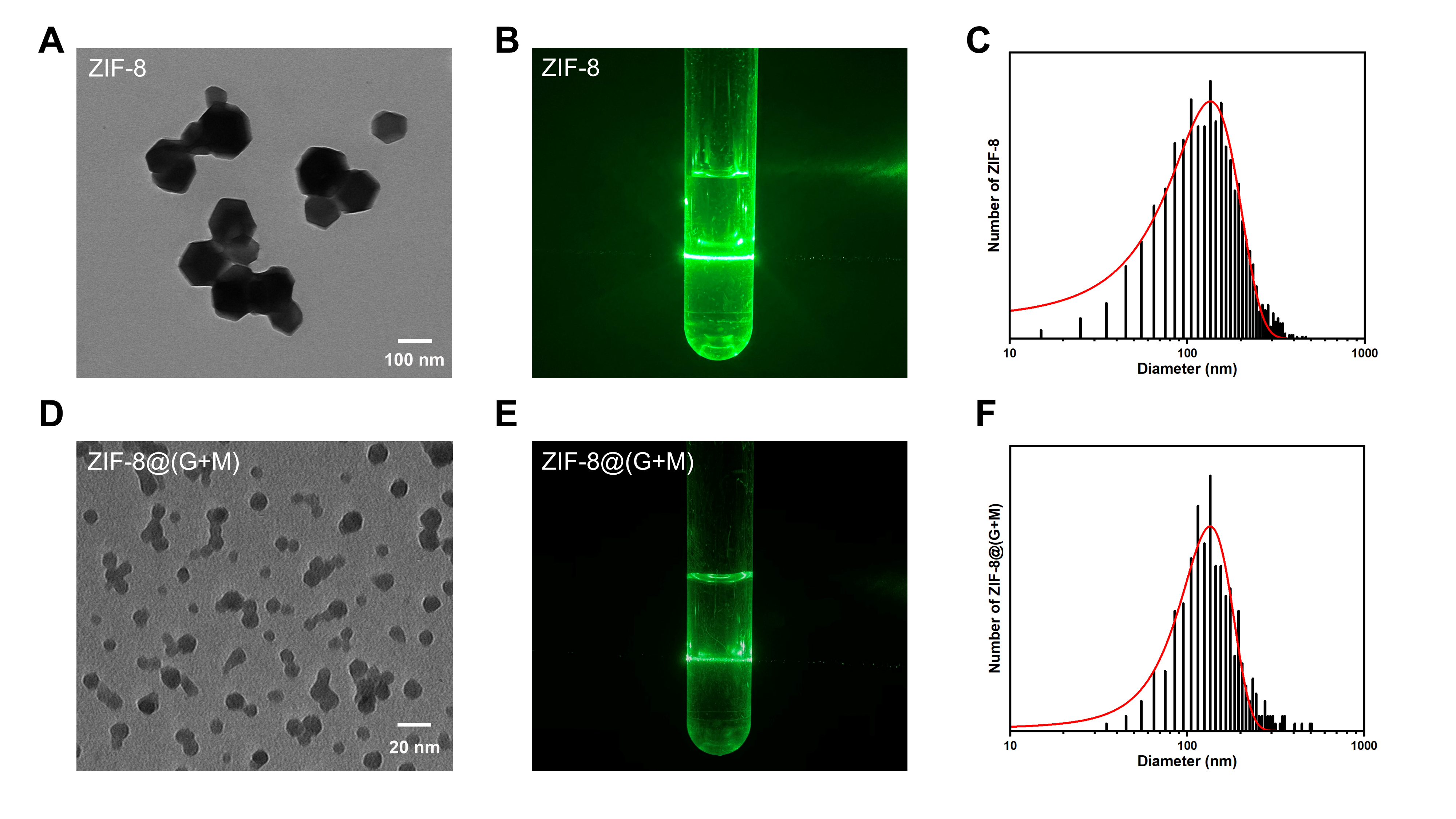

Supplement: Supplementary file 1 — Additional file 1. [file 13046_2022_2372_MOESM1_ESM.zip › Supplementary Figures/Fig. S1.tif]

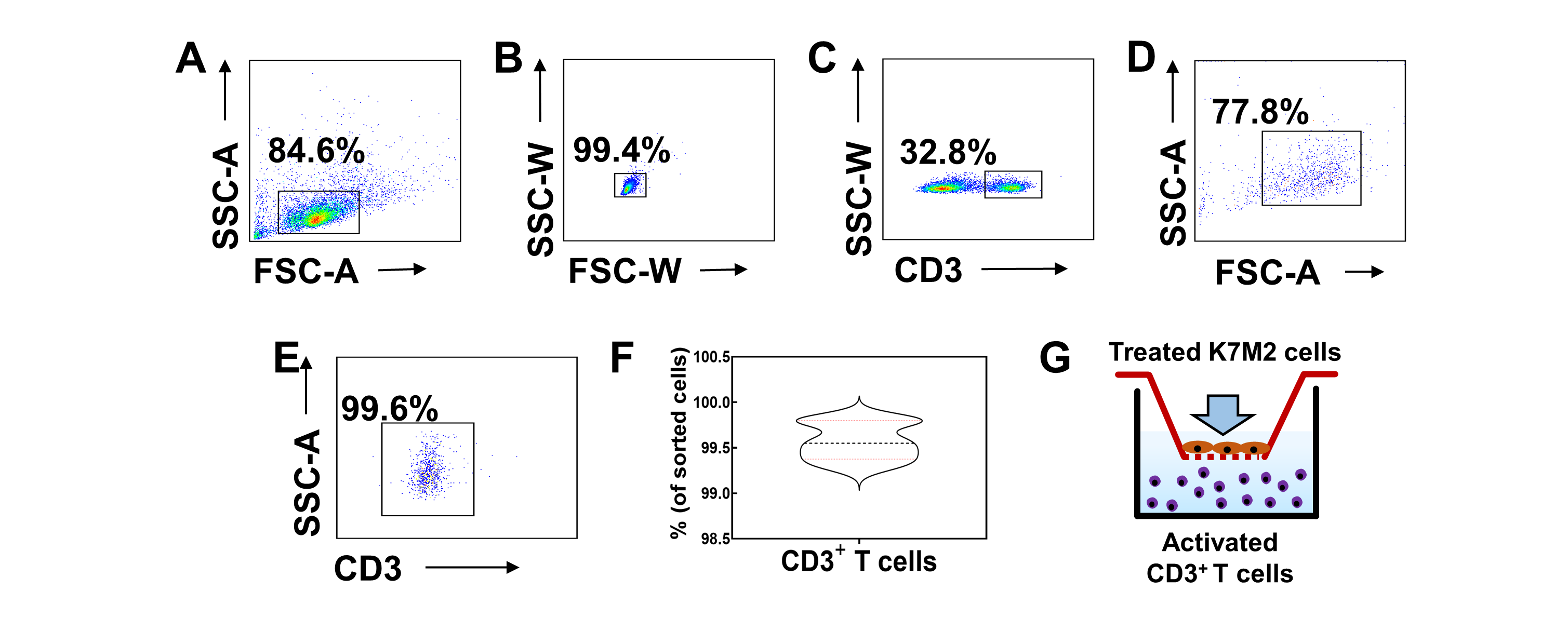

Supplement: Supplementary file 1 — Additional file 1. [file 13046_2022_2372_MOESM1_ESM.zip › Supplementary Figures/Fig. S2.tif]

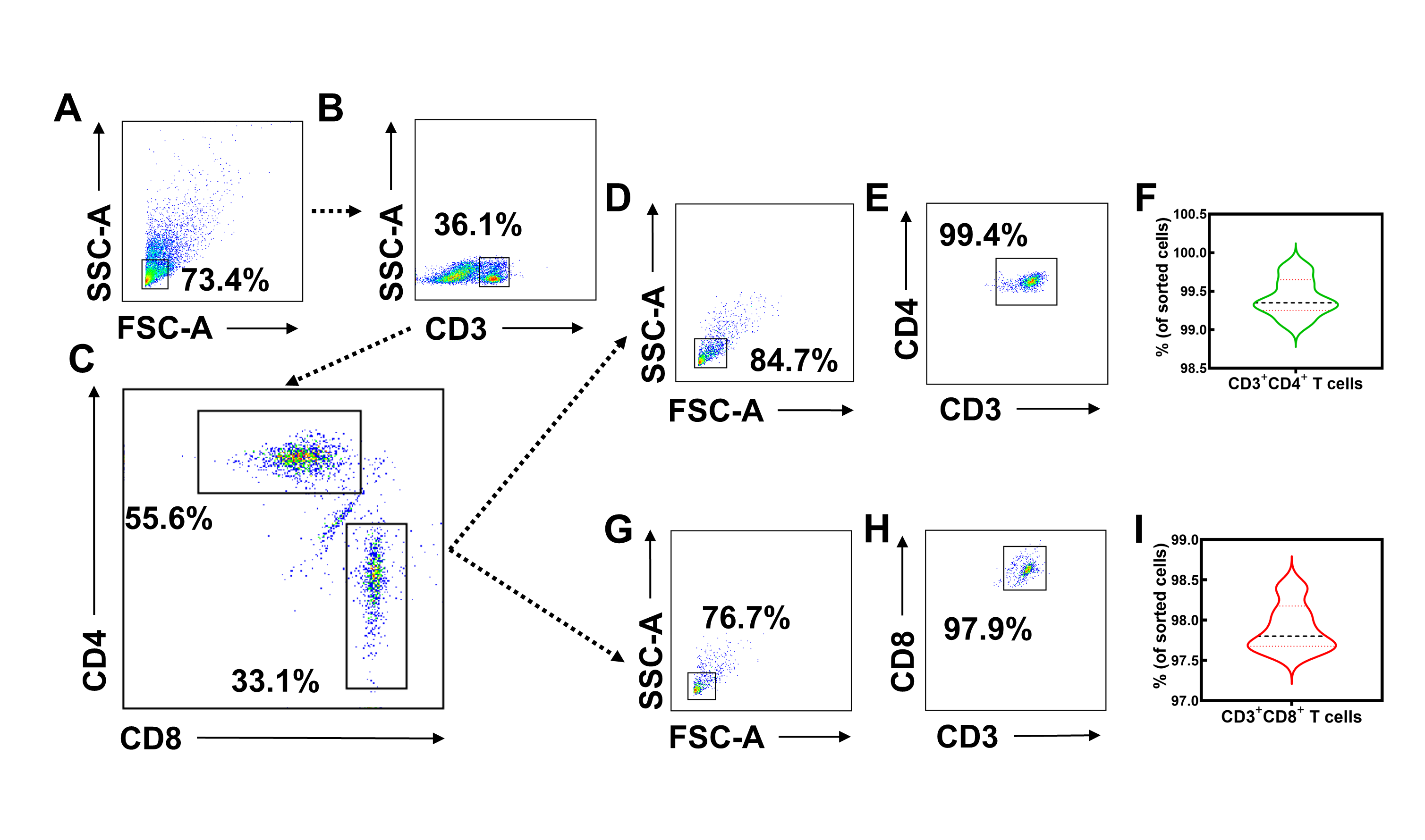

Supplement: Supplementary file 1 — Additional file 1. [file 13046_2022_2372_MOESM1_ESM.zip › Supplementary Figures/Fig. S3.tif]

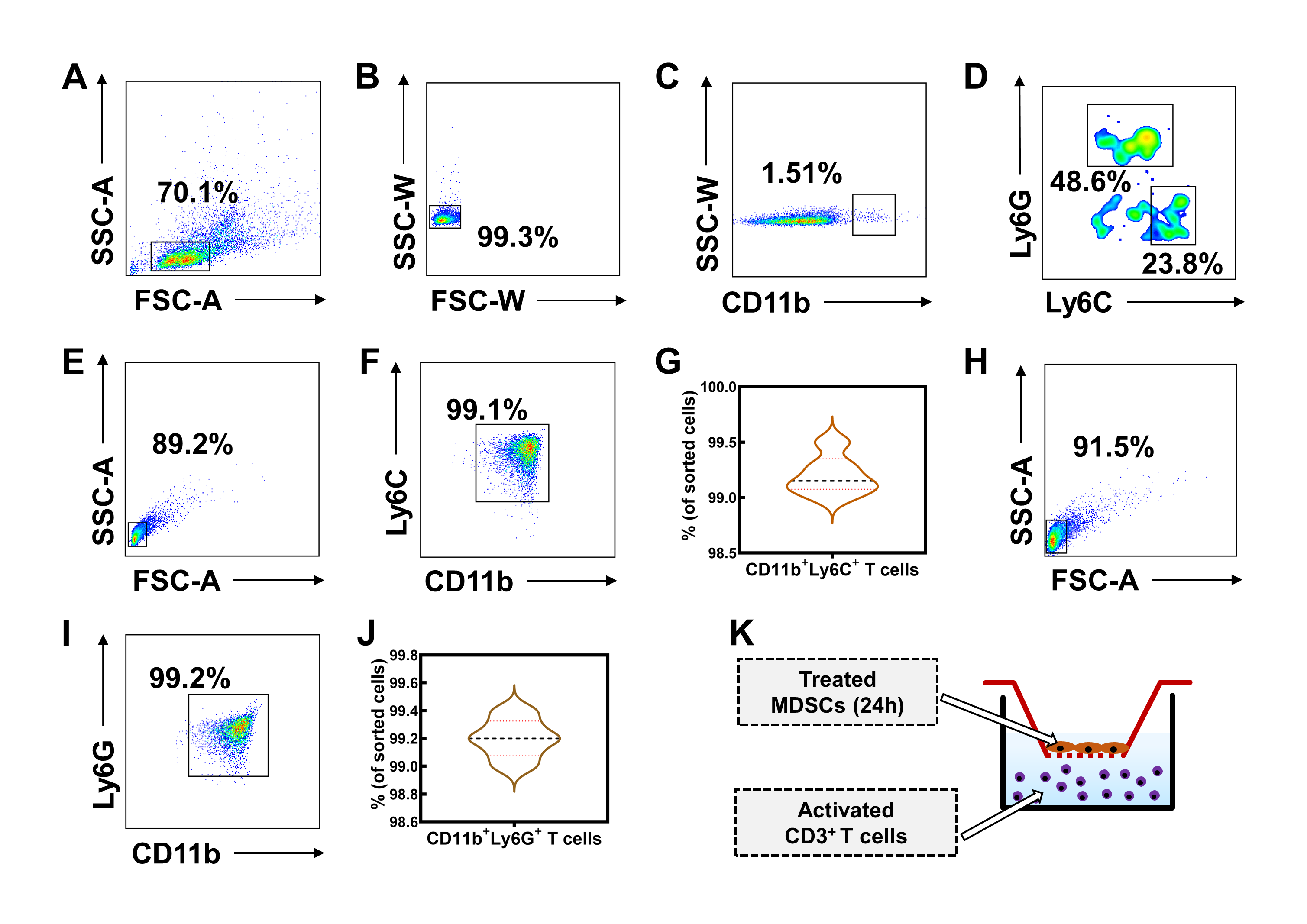

Supplement: Supplementary file 1 — Additional file 1. [file 13046_2022_2372_MOESM1_ESM.zip › Supplementary Figures/Fig. S4.tif]

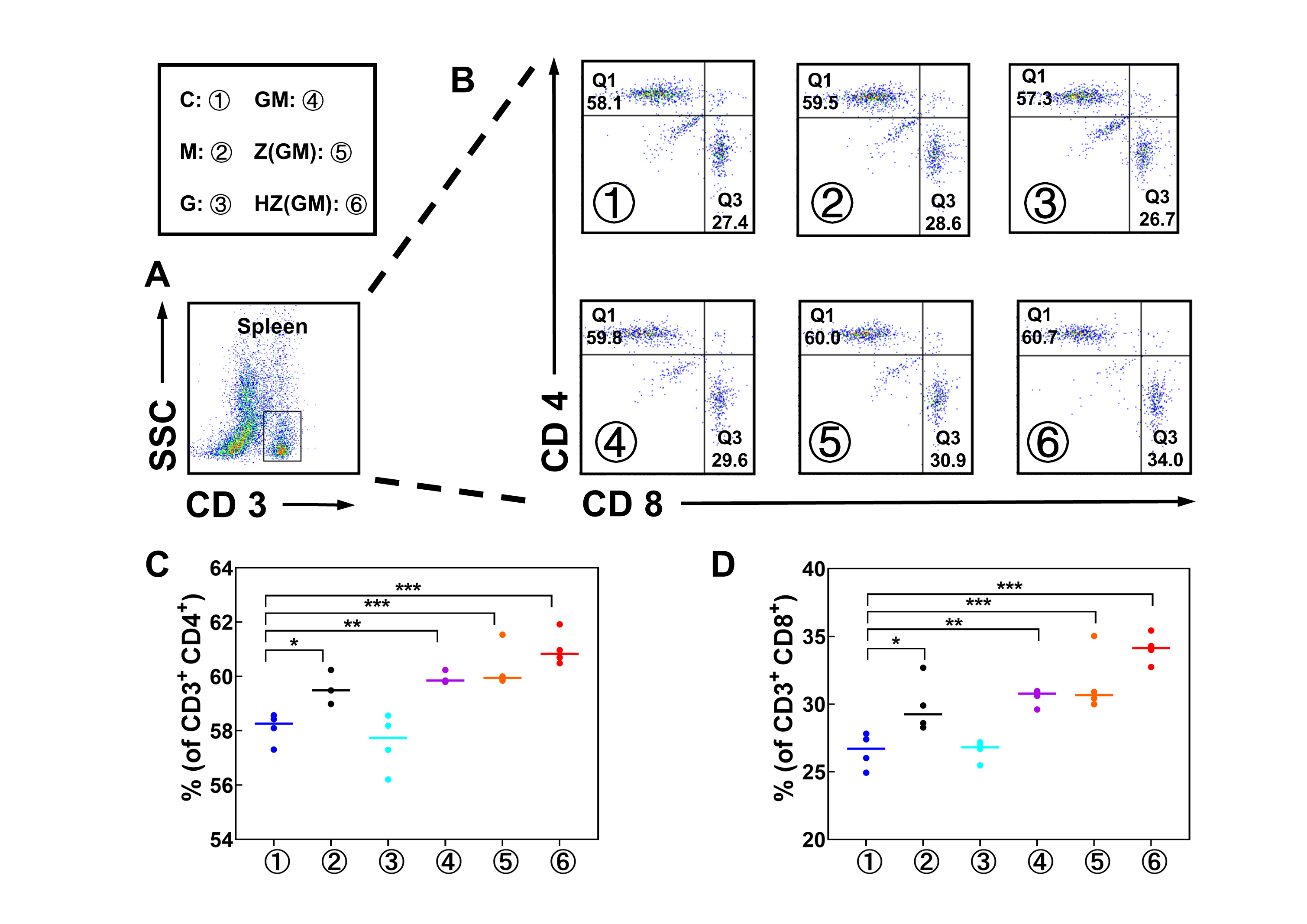

Supplement: Supplementary file 1 — Additional file 1. [file 13046_2022_2372_MOESM1_ESM.zip › Supplementary Figures/Fig. S5.tif]
